# Australian long-finned pilot whales (*Globicephala melas*) emit stereotypical, variable, biphonic, multi-component, and sequenced vocalisations, similar to those recorded in the northern hemisphere

**DOI:** 10.1038/s41598-020-74111-y

**Published:** 2020-11-26

**Authors:** Rachael Courts, Christine Erbe, Rebecca Wellard, Oliver Boisseau, K. Curt Jenner, Micheline-N. Jenner

**Affiliations:** 1 Centre for Marine Science and Technology, Curtin University, Perth, WA 6102 Australia; 2 Project ORCA, Perth, WA 6026 Australia; 3 Song of the Whale Research Team, Marine Conservation Research, 94 High Street, Kelvedon Essex, CO5 9AA UK; 4 Centre for Whale Research (WA) Inc., PO Box 1622, Fremantle, WA 6959 Australia

**Keywords:** Ecology, Zoology, Ecology, Environmental sciences, Ocean sciences

## Abstract

While in the northern hemisphere, many studies have been conducted on the vocal repertoire of long-finned pilot whales (*Globicephala melas*), no such study has been conducted in the southern hemisphere. Presented here, is the first study on the vocalisations of long-finned pilot whales along the southern coast of mainland Australia. Multiple measures were taken of 2028 vocalisations recorded over five years in several locations. These vocalisations included tonal sounds with and without overtones, sounds of burst-pulse character, graded sounds, biphonations, and calls of multiple components. Vocalisations were further categorised based on spectrographic features into 18 contour classes. Altogether, vocalisations ranged from approximately 200 Hz to 25 kHz in fundamental frequency and from 0.03 s to 2.07 s in duration. These measures compared well with those from northern hemisphere pilot whales. Some call types were almost identical to northern hemisphere vocalisations, even though the geographic ranges of the two populations are far apart. Other call types were unique to Australia. Striking similarities with calls of short-finned pilot whales (*Globicephala macrorhynchus*) and sometimes sympatric killer whales (*Orcinus orca*) were also found. Theories for call convergence and divergence are discussed.

## Introduction

Cetaceans rely on sound in support of various life functions. They communicate acoustically during social interactions, foraging, mating, and rearing of young. Acoustic communication serves to coordinate group behaviour and maintain group cohesion^1^. Additionally, odontocetes use bio-sonar to navigate and capture prey^2^. The acoustic repertoire of odontocetes has been described as complex, and vocalisations can vary on a species, population, or individual level^3–5^.

Odontocetes, in general, emit three types of vocalisations: whistles, burst-pulse sounds, and clicks^6^. Whistles are continuous tonal sounds, consisting of a fundamental contour with or without harmonically related overtones. They are likely used to encode group or individual identity^7,8^. Burst-pulse sounds are bursts of brief pulses emitted at high repetition rates. They have been shown to encode group identity and to mediate group behaviours^8–10^, while also playing a role in specific behavioural displays such as aggression^11^. Clicks are brief pulses, typically emitted in trains, and primarily used for echolocation during navigation and foraging^2^. Some odontocetes also communicate with clicks^12–14^.

Some odontocete species further produce vocalisations that have aspects of both whistles and burst-pulse sounds. Graded vocalisations gradually transition from whistles to burst-pulse sounds, or vice versa^15,16^. Biphonations refer to the production of two distinct sounds simultaneously, such as two simultaneous whistles or a whistle with a burst-pulse sound^17,18^. Multi-component vocalisations comprise two or more components in immediate succession, without any gap in time. These components can be of whistle or burst-pulse type, or, are biphonations. Multi-component vocalisations may exhibit abrupt changes in pulse repetition rate when two burst-pulse sounds are joined^19–21^.

Whistles and burst-pulse sounds differ on a species, population, and sometimes individual level. Species-specific vocalisations have been recorded worldwide, for example in the western North Atlantic^22^ or around Australia^6^. Population-specific vocalisation variations have been well documented in North Pacific killer whales^3^. Individually distinctive signature whistles have been identified in several delphinids^5,23^. Burst-pulse sounds may also convey identity^24^. Whistles and burst-pulse sounds of the same species may further change with geographic region, habitat, and ecological factors^4,25^, and in response to ambient noise^26,27^.

Long-finned pilot whales (*Globicephala melas*) are the second largest species in the delphinid family, and are distributed in two disjunct populations, with no overlap in home range^28,29^. Northern hemisphere long-finned pilot whales occur from the eastern seaboard of the USA to the Mediterranean Sea^30^ at latitudes between approximately 30° and 70° N, with a population size of several hundred thousand individuals^31^. Southern hemisphere long-finned pilot whales occur along the southern coastlines of South Africa, South America, New Zealand, and Australia^32,33^. Population estimates of long-finned pilot whales in the southern hemisphere do not exist^32,34^. The acoustic repertoire of northern hemisphere long-finned pilot whales has been described as highly diverse, repetitive, and physically complex^19,35–37^. To date, no data have been reported on long-finned pilot whale sound usage in the southern hemisphere^6^.

Quantification of a species’ or population’s acoustic repertoire is necessary to develop passive acoustic monitoring (PAM) programs. Such programs can be effective for studying distribution, abundance, behaviour, communication, and the potential effects of anthropogenic factors such as noise^38^. For the first time, we quantify the vocalisations produced by long-finned pilot whales off southern Australia and highlight similarities and differences of the vocalisations of northern hemisphere long-finned pilot whales.

## Results

A total of 2028 long-finned pilot whale vocalisations were recorded at sufficient quality for analysis. In total, 18 vocalisation classes were found based on similarity in contour, frequency, and duration. The inter-observer reliability test produced a Fleiss’ kappa of 0.63 indicating ‘substantial agreement’.

These 2028 vocalisations consisted of 2148 components. Overall, the mean duration was 0.42 ± 0.28 s. The mean minimum frequency was 4.3 ± 2.7 kHz and mean maximum frequency was 5.9 ± 3.5 kHz (Table 1).

**Table 1:** Measurement summary of the lowest contours of all vocalisation components (*n* = 2148).

	Dur (s)	Min f (Hz)	Max f (Hz)	Start f (Hz)	End f (Hz)	Extr (n)	Infl (n)	Step (n)	Overtone (n)
Mean	0.42	4288	5936	4826	5362	0.3	0.2	0.2	1.2
Standard deviation	0.28	2719	3462	2864	3485	0.6	0.7	0.8	2.9
Minimum	0.03	219	693	405	310	0.0	0.0	0.0	0.0
Maximum	2.07	14,685	24,787	18,270	24,781	7.0	9.0	10.0	50.0
10th Percentile	0.12	1545	2497	1972	1968	0.0	0.0	0.0	0.0
25th Percentile	0.19	2443	3340	2722	2799	0.0	0.0	0.0	0.0
50th Percentile	0.37	3464	4917	3980	4348	0.0	0.0	0.0	0.0
75th Percentile	0.60	5599	8012	6363	7344	0.0	0.0	0.0	1.0
90th Percentile	0.81	8103	10,572	8923	9898	1.0	1.0	0.0	4.0

Duration (*Dur*) [s], minimum frequency (*Min f*) [Hz], maximum frequency (*Max f*) [Hz], start frequency (*Start f*) [Hz], end frequency (*End f*) [Hz], number of local extrema (*Extr*) [n], number of inflection points (*Infl*) [n], number of steps (*Step*) [n] and number of overtones [n].

**Figure 1:**
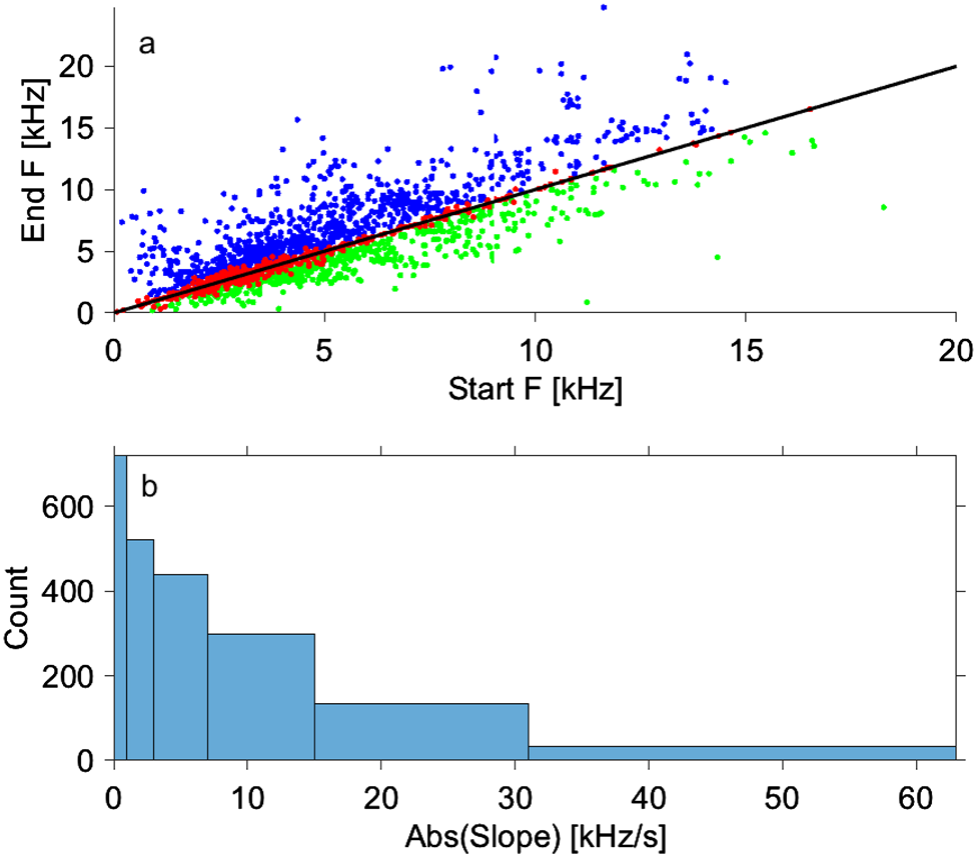
(**a**) Scatter plot of end frequency versus start frequency showing overall upsweeping contours (blue) above the diagonal and downsweeping contours (green) below. Vocalisations classified as flat (red) hug the diagonal. (**b**) Histogram of the absolute of the slope in logarithmically increasing bins with edges at 0, 1, 3, 7, 15, 31, and 63 kHz.

Figure 1 shows a scatter plot of end frequency versus start frequency and a histogram of the absolute of the slope of all 2148 fundamental contours. Under the criterion of an allowable slope of ± 1 kHz/s, 719 contours were considered flat, 867 upsweeping, and 562 downsweeping.

### Vocalisation classes

The following sections describe 18 vocalisation classes.

#### Class-1

**Figure 2:**
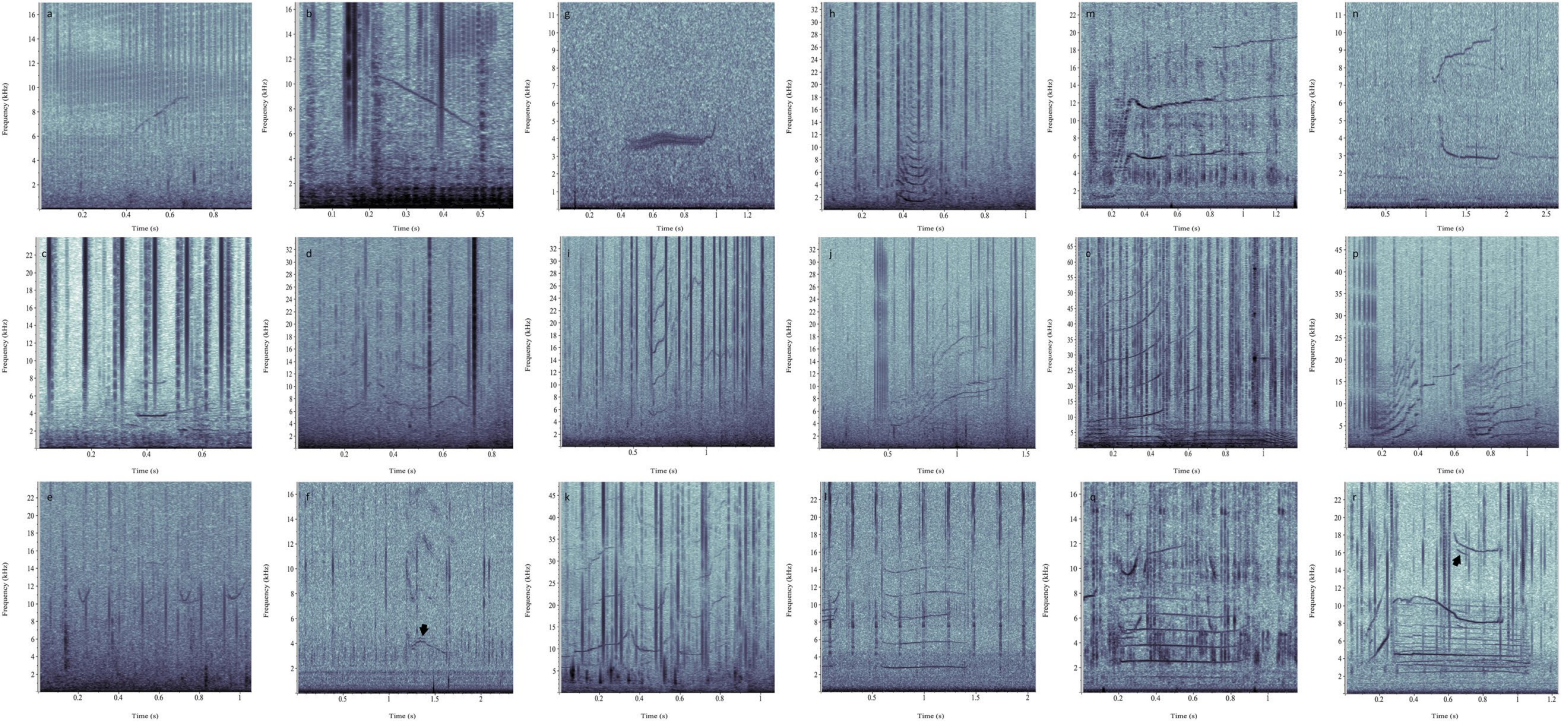
Spectrograms of 18 contour classes: (**a**) Class-1, (**b**) Class-2, (**c**) Class-3, (**d**) Class-4, (**e**) Class-5, (**f**) Class-6, arrow points to sideband, (**g**) Class-7, (**h**) Class-8, (**i**) Class-9, (**j**) Class-10, (**k**) Class-11, (**l**) Class-12, (**m**) Class-13, (**n**) Class-14, (**o**) Class-15, (**p**) Class-16, (**q**) Class-17, (**r**) Class-18, arrow points to sideband. Spectrogram settings: 47-Hz resolution, Hann window, 90% overlap.

Class-1 contained single-component, frequency-modulated tones that lasted, on average, 0.40 s (s.d. = 0.25 s) and appeared in spectrograms as an overall upsweep (Fig. 2a). Frequency ranged, on average, from 4707 Hz (s.d. = 2468 Hz) to 6716 Hz (s.d. = 3143 Hz) (Supplementary Table S1). The average slope for Class-1 was 7406 Hz/s. Additionally, 32% of all Class-1 vocalisations showed harmonic overtones. This vocalisation was recorded 654 times across all locations.

#### Class-2

Class-2 lasted, on average, 0.43 s (s.d. = 0.28 s) and contained single-component, frequency-modulated tones that appeared in spectrograms as an overall downsweep (Fig. 2b). Frequency ranged from 4090 Hz (s.d. = 2286 Hz) to 5454 Hz (s.d. = 2747 Hz) (Supplementary Table S2). The average slope for Class-2 was − 4917 Hz/s. Additionally, 28% of vocalisations had harmonic overtones. Class-2 vocalisations occurred 488 times across all locations and years. A maximum of 10 steps was found in this class.

#### Class-3

Vocalisations of Class-3 lasted approximately 0.44 s (s.d = 0.31 s) and were identified as single-component tones without frequency modulation (i.e., flat contour) (Fig. 2c). Class-3’s frequency ranged from 3806 Hz (s.d. = 2329 Hz) to 4331 Hz (s.d. = 2456 Hz) (Supplementary Table S3). A slope of − 1000 to + 1000 Hz/s was accepted. Additionally, 31% of these vocalisations had harmonic overtones. This class was recorded 316 times across all locations and years.

#### Class-4

Class-4 contained single-component frequency-modulated tones that lasted, on average, 0.61 s (s.d. = 0.37 s) and appeared in spectrograms as an overall wavy (‘sine’) contour (Fig. 2d). The frequency ranged from 5346 Hz (s.d. = 3004 Hz) to 7524 Hz (s.d. = 3636 Hz) (Supplementary Table S4). Out of all Class-4 vocalisations, 32% showed harmonic overtones. This vocalisation was recorded 58 times across all locations and years.

#### Class-5

Class-5 consisted of 0.32 s (s.d. = 0.27 s) single-component, frequency-modulated tones of convex (i.e., U-shaped) contour (Fig. 2e). Average frequency ranged from 4569 Hz (s.d. = 3199 Hz) to 5850 Hz (s.d. = 3609 Hz) (Supplementary Table S5). Out of the 159 Class-5 vocalisations recorded, 37% showed harmonic overtones. Class-5 occurred in all locations and years.

#### Class-6

Vocalisations of Class-6 lasted, on average, 0.38 s (s.d = 0.29 s). These were single-component, frequency-modulated tones that appeared as an overall concave (i.e., inverted-U) contour in spectrograms (Fig. 2f). Class-6’s frequency ranged from 4552 Hz (s.d. = 2715 Hz) to 5711 Hz (s.d. = 3004 Hz) (Supplementary Table S6). Some of the vocalisations had steps. Out of the 111 Class-6 vocalisations recorded, 18% had harmonic overtones. Some of Class-6 also showed sidebands indicative of amplitude modulation (Fig. 2f). These vocalisations were recorded in all locations and years.

#### Class-7

Class-7 consisted of a 0.63-s (s.d. = 0.12 s) amplitude-modulated tone (Fig. 2g). Class-7 frequency ranged from 3202 Hz (s.d. = 238 Hz) to 5015 Hz (s.d. = 503 Hz) (Supplementary Table S7). This vocalisation occurred 28 times, in the GAB in 2013.

#### Class-8

Class-8 vocalisations were brief: 0.31 s (s.d. = 0.21 s). All of these vocalisations exhibited many (> 10) overtones in spectrograms and are thus considered to be of burst-pulse type^39^. The pulse-repetition rate was variable, so that these vocalisations appeared in spectrograms as flat, downsweep, U-shaped or inverted-U-shaped contours (Fig. 2h). Frequency ranged from 1830 Hz (s.d. = 1180 Hz) to 2914 Hz (s.d. = 1361 Hz) (Supplementary Table S8). Vocalisations of this class were recorded 73 times across most locations and years, however not in the BSB in 2015.

#### Class-9

Class-9 vocalisations were also brief: 0.43 s (s.d. = 0.26 s). They appeared in spectrograms as upsweeps (Fig. 2i). Some of these vocalisations had contours that plateaued and ended flat. Frequency, on average, ranged from 2243 Hz (s.d. = 1106 Hz) to 4741 Hz (s.d. = 2445 Hz) (Supplementary Table S9). Class-9 occurred 19 times across most locations and years, however, was not recorded in the BSB in 2015 nor 2016.

#### Class-10

Class-10 had similar contours as Class-9 (upsweep and plateaued upsweep) but were longer in duration: 0.69 s (s.d. = 0.16 s) (Fig. 2j). Class-10’s average frequency ranged from 1645 Hz (s.d. = 953 Hz) to 7842 Hz (s.d. = 2728 Hz) (Supplementary Table S10). These vocalisations were recorded 11 times in BSB, seven times in 2014 and four times in 2016.

#### Class-11

Class-11 lasted on average 0.96 s (s.d. = 0.22 s) and appeared in spectrograms (Fig. 2k) as an overall sine contour. Frequency ranged from 4218 Hz (s.d. = 1893 Hz) to 9036 Hz (s.d. = 4806 Hz) (Supplementary Table S11). Compared to Class-4, Class-11 was longer in duration and had many more overtones in spectrograms. This vocalisation was recorded three times, only in the BSB (2014, 2016, and 2017).

#### Class-12

Class-12 had a duration average of 0.82 s (s.d. = 0.27 s) and appeared in spectrograms as flat by the above definition of slope changes in the fundamental of less than ± 1 kHz/s (Fig. 2l). Class-12’s frequency ranged from 1783 Hz (s.d. = 860 Hz) to 2621 Hz (s.d. = 1066 Hz) (Supplementary Table S12). Compared to Class-3, Class-12 had many more overtones. Compared to Class-8, Class-12 was longer in duration. These vocalisations were recorded 13 times, most recorded in the GAB in 2013 and 2016, and recorded twice in the BSB in 2016 and 2017.

#### Class-13

Class-13 vocalisations started with a steep upsweep, had a local maximum, and transitioned into a mildly upsweeping tone (Fig. 2m). Overall duration was 1.16 s (s.d. = 0.13 s). Frequency ranged from 567 Hz (s.d. = 102 Hz) to 5134 Hz (s.d. = 1529 Hz) (Supplementary Table S13). The initial upsweep had many overtones, exhibiting burst-pulse characteristics. As the contour flowed without steps or gaps from the upsweep, through the maximum, into the final upsweep, this vocalisation was graded, changing smoothly from burst-pulse to tonal character. This vocalisation was recorded three times in the GAB in 2016.

#### Class-14

Class-14 vocalisations were biphonations consisting of two separately modulated sounds. Part 1 had an average duration of 0.61 s (s.d. = 0.18 s) with a frequency from 2364 Hz (s.d. = 866 Hz) to 3888 Hz (s.d. = 1548 Hz). Part 1 was a downsweep, sometimes showing many overtones (Fig. 2n). Part 2 had a shorter duration than Part 1: 0.44 s (s.d. = 0.21 s). Part 2’s contour was an upsweep that ranged in frequency from 8502 Hz (s.d. = 2707 Hz) to 11,033 Hz (s.d. = 2638 Hz). Class 14 was recorded 48 times, only occurring in the GAB in 2013.

#### Class-15

Vocalisations of Class-15 were biphonations. Part 1 ranged in frequency from 715 Hz (s.d. = 421 Hz) to 4008 Hz (s.d. = 890 Hz) (Supplementary Table S15). Part 1 lasted on average 0.89 s (s.d. = 0.11 s) and appeared in the spectrogram (Fig. 2o) as a contour that started flat, then rose, plateaued, then decreased and ended flat. This part was longer in duration than the second part of this biphonation. Part 2 lasted on average 0.43 s (s.d. = 0.13 s). In the spectrogram (Fig. 2o) the contour had a step towards higher frequency at 0.20 s. On average, the frequency ranged from 8625 Hz (s.d. = 1795 Hz) to 17,230 Hz (s.d. = 3754 Hz) (Supplementary Table S15). This vocalisation was recorded 16 times and was only recorded in the BSB in 2017. Note that the 30-kHz tone in Fig. 2o is from an echosounder and not part of this vocalisation.

#### Class-16

Class-16 vocalisations comprised three successive parts: an upsweep with many overtones, followed by a stepped tone and then another component with many overtones (Fig. 2p). Part 1 appeared in spectrograms as an upsweep of, on average, 0.29 s (s.d. = 0.08 s), with a frequency ranging from 1059 Hz (s.d. = 396 Hz) to 5627 Hz (s.d. = 1027 Hz) (Supplementary Table S16). Given the many overtones, this component was likely a burst-pulse sound of increasing pulse repetition rate (appearing as upsweeping in the spectrogram). Part 2 was a stepped-up tone which lasted, on average, 0.24 s (s.d. = 0.05 s). Part 2 frequency ranged from 11,781 Hz (s.d. = 1592 Hz) to 17,873 Hz (s.d. = 1638 Hz) (Supplementary Table S16). Part 3 lasted on average 0.50 s (s.d. = 0.17 s) and appeared in spectrograms as a contour that began flat, then rose and plateaued. Given the many overtones, this was likely another burst-pulse sound. Part 3 was longer in duration than Parts 1 and 2 and, on average, ranged in frequency from 978 Hz (s.d. = 408 Hz) to 3675 Hz (s.d. = 957 Hz). This vocalisation was recorded 13 times and was only recorded in the BSB in 2016.

#### Class-17

Vocalisations of Class-17 were biphonations consisting of three parts (Fig. 2q). Part 1 was a 0.14 s (s.d. = 0.01 s) tone of convex contour with a frequency between 4600 Hz (s.d. = 263 Hz) and 6010 Hz (s.d. = 674 Hz) (Supplementary Table S17). Part 2 was a 0.61 s (s.d. = 0.16 s) flat contour with many overtones; the fundamental had an average frequency from 1245 Hz (s.d. = 419 Hz) to 1718 Hz (s.d. = 478 Hz) (Supplementary Table S17). Part 3 was a 0.25 s (s.d. = 0.06 s) upsweep with an average frequency from 8901 Hz (s.d. = 768 Hz) to 12,160 Hz (s.d. = 203 Hz) (Supplementary Table S17). Parts 1 and 3 occurred in succession but at the same time as Part 2. This vocalisation was recorded eight times, only in the GAB in 2016. Convex tones like Part 1 also occurred singularly and were classified as Class-5. Flat vocalisations like Part 2 also existed as single-component vocalisations (Class-12). Part 3 was similar to Class-1. The three parts were considered one vocalisation, because they had similar received levels, and their frequency and duration measurements, and relative timing within the vocalisation were very similar in the two occurrences.

#### Class-18

Vocalisations of Class-18 comprised three parts (Fig. 2r). Part 1 was a 0.14 s (s.d. = 0.03 s) upsweep with a frequency between 3781 Hz (s.d. = 501 Hz) and 7089 Hz (s.d. = 915 Hz) (Supplementary Table S18). Part 2 was a 0.79 s (s.d. = 0.14 s) flat contour with many overtones; the fundamental had an average frequency from 2232 Hz (s.d. = 266 Hz) to 2827 Hz (s.d. = 354 Hz) (Supplementary Table S18). Part 3 was a 0.62 s (s.d. = 0.18 s) downsweep that showed, on average, a frequency between 8614 Hz (s.d. = 1526 Hz) and 11,907 Hz (s.d. = 1591 Hz) (Supplementary Table S18). Parts 2 and 3 occurred simultaneously, even though Part 2 was longer in duration than Part 3. Some Part 3’s of Class-18 contained sidebands (Fig. 2r) indicative of amplitude modulation. This vocalisation was recorded seven times and was only recorded in the GAB in 2016.

### Vocalisation sequences

**Figure 3:**
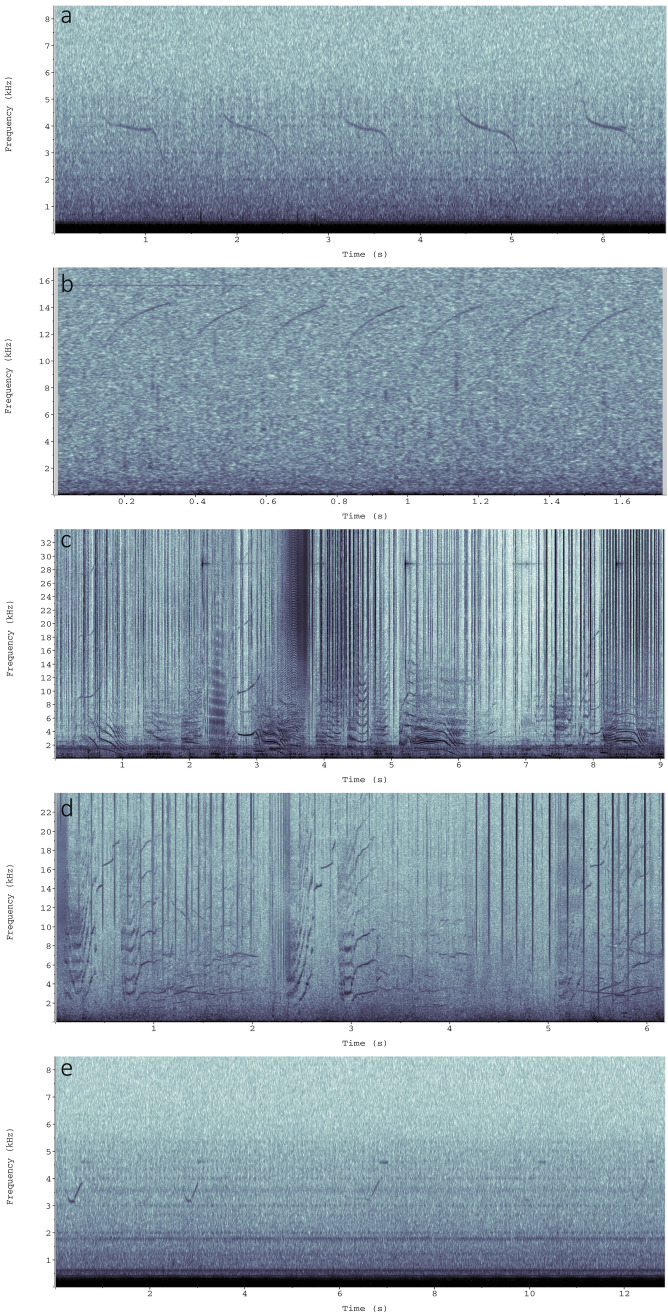
Spectrograms of repeated vocalisations and one example of duetting: (**a**) repeated downsweeps (Class 2), (**b**) repeated upsweeps (Class 1), (**c**) repeated biphonation (Class 15), (**d**) repeated three-part vocalisation (Class 16), and (**e**) duet of convex (Class 5) and constant vocalisations (Class 3). Spectrogram settings: 47-Hz resolution, Hann window, 90% overlap.

Spectrogram examples of vocalisation sequences are shown in Fig. 3. Supplementary Fig. S1 shows histograms of the percentages of instances that any vocalisation class followed each of the classes. The results are also summarised in Table 2. While some classes were more common than others (with Class-1 making up 32% of the vocalisations, Class-2 24%, and Class-3 16%), these were not always the most common followers. Vocalisations of classes 1, 2, 8, 13, 14, 15, and 16 were more often followed by a vocalisation of the same class than by any other class. This was particularly noticeable for classes 13–16 with probabilities of being followed by the same class exceeding 50%.

**Table 2:** Number of times each class was recorded, the class by which it was most commonly followed, the probability of detecting a vocalisation from the same class in immediate succession, and the median time between vocalisations of the same class.

Class	No. times recorded	Percentage of calls	Most commonly followed by	Likelihood of being immediately followed by same class [%]	Median time to same class [s]
C 1	654	32.2	C 1	45	5.0
C 2	488	24.1	C 2	44	4.7
C 3	316	15.6	C 1, then C 3	26	11.5
C 4	58	2.9	C 2	7	61.5
C 5	159	7.8	C 1, then C 2, then C 5	19	15.2
C 6	111	5.5	C 1, then C 2, then C 6	15	47.8
C 7	28	1.4	C 1, then C 7	21	27.3
C 8	73	3.6	C 8	40	15.8
C 9	19	0.9	C 1	6	41.6
C 10	11	0.5	C 1	9	7.7
C 11	3	0.1	C 1	0	0.0
C 12	13	0.6	C 2 and C 6	11	510.1
C 13	3	0.1	C 13	100	0.0
C 14	48	2.4	C 14	65	7.1
C 15	16	0.8	C 15	69	5.6
C 16	13	0.6	C 16	54	4.4
C 17	8	0.4	C 2, then C 17	20	0.6
C 18	7	0.3	C 1	0	0.0

Sequences involving two different vocalisations were also noted. The spectrogram in Fig. 3e shows a sequence of five U-shaped and five constant wave contours. The relative received levels change from instance to instance, whereby the animal making the U-shaped vocalisation is closer to the recorder at first, but the animal making the constant wave is closer at the third instance.

## Discussion

This article provides the first description of long-finned pilot whale vocalisations recorded off southern Australia. The repertoire comprised tonal sounds with and without overtones, sounds of burst-pulse character, graded vocalisations, biphonations, and multi-component vocalisations. While vocalisations were grouped into 18 classes in order to describe their spectral and temporal features, these classes do not imply any specific function, and animals as well as other human observers may well group sounds differently. Setting criteria for separate spectrographic classes is particularly difficult when animals emit graded vocalisations, as documented for beluga whales (*Delphinapterus leucas*^15^), false killer whales (*Pseudorca crassidens*^16^), killer whales (*Orcinus orca*^21^), and northern hemisphere long-finned pilot whales^40^.

‘Gradation’ may appear in different ways. Vocalisations may change gradually in spectrographic measurements such as frequency and duration. This is obvious from the overlapping frequency and duration measurements of Classes-3, 8, and 12, for example, all of which had flat contours. However, Class-3 had the highest mean frequency measurements and fewer or no overtones. Class-8 had the shortest mean durations and the most overtones. Also, frequency modulation may change gradually (see the variants in Class-8), which has been well illustrated by Taruski^40^. Finally, vocalisations may change gradually from tonal, to amplitude-modulated (e.g., appearance of sidebands in Classes-6 and 18), to pulsed^16^.

Overall, Australian long-finned pilot whale tonal frequencies were comparable to those reported from northern hemisphere long-finned pilot whales, showing similarities in contour, fundamental frequency, and duration. Mean minimum frequencies ranged from 2.8 kHz to 3.5 kHz in the North Atlantic^19^, Western North Atlantic^22^, and Mediterranean^41^. Mean maximum frequencies ranged from 4.7 kHz to 6.4 kHz in the northern hemisphere^19,22,41^. Therefore, mean minimum frequency of southern-Australian long-finned pilot whales (4.2 kHz) was almost 1 kHz higher than northern populations. Three unique vocalisations were recorded from southern-Australian long-finned pilot whales, which have not been reported for the species. These are the multi-component classes 15, 17 and 18, all of which include a biphonation (Fig. 2o,q,r).

Differences in frequency ranges may arise from physiological, behavioural, or environmental factors^41–47^. Frequency dissimilarity may be explained by body size, whereby smaller animals produce higher frequencies compared to larger animals that emit lower frequencies. Such a relationship has been documented in odontocetes^43^, but evidence is lacking demonstrating that Australian long-finned pilot whales are smaller in body size than northern hemisphere long-finned pilot whales. Previously, associations have been made between vocalisation type and behaviour, whereby simple vocalisations are emitted during resting behaviours, and complex vocalisations during surface-active behaviours^48^. Therefore, it is possible that differences in vocalisation parameters indicate different behavioural states in long-finned pilot whales at the time of recording. Time of day may also be an influencing factor, as daylight significantly correlates with behavioural events^46^. Whistles from long-finned pilot whales in noisier environments have shown higher frequencies than those from environments with low noise^41^. However, ambient noise in southern Australia might be lower due to less shipping than in the North Atlantic and Mediterranean^49^, predicting lower frequencies in southern Australian long-finned pilots; yet this was not observed. Finally, sample sizes differed between studies and the full repertoire might not have been captured by all studies, affecting reported frequency ranges.

Considering other parameters measured, only 17% of all tonal sounds had inflection points, with a maximum of nine inflection points per contour. Typically, vocalisations with zero inflection points are considered ‘simple’, and vocalisations with more inflection points are considered ‘complex’^19,40,48^, suggesting that Australian long-finned pilot whales, like northern hemisphere long-finned pilot whales, have a mixture of simple and complex vocalisations.

Vocalisations from classes 1–6 were recorded across all seven study site and year combinations and thus unaffected by animal behaviour, number of animals, or recording duration. These are the simplest classes (straight upsweeps, downsweeps, flat tones, sinusoidals, convex and concave contours). The largest number and variety of complex vocalisations was observed in the GAB in 2016, when long-finned pilot whales displayed travelling behaviour. Similar findings have been reported in North Atlantic pilot whales, where a greater number of vocalisation classes (including complex vocalisations) were recorded when pilot whales were travelling with a population spread over a large area^48^. At the GAB in 2016, the southern Australian long-finned pilot whales may have been spread out over a larger area than in other years.

Noteworthy similarities of specific vocalisation contours were observed amongst the Australian and northern hemisphere populations. Most of the Australian whistles classified as Class-3 (Fig. 2c), were strikingly similar to North Atlantic ‘S1-level frequency’, with fundamentals approximately ranging from 3 to 5 kHz, durations of 0.45 s to 2.0 s and harmonic overtones^48^. Class-2 (Fig. 2b) and Class-1 (Fig. 2a) largely matched North Atlantic ‘S2 falling frequency’ and ‘S3 rising frequency’, respectively, showing durations from 0.4 s to 3.0 s. However, Class-2 and Class-1 had lower mean minimum frequencies than the vocalisations from the North Atlantic^48^. Class-6 (Fig. 2f) and Class-5 (Fig. 2e) showed similarity in overall contour to North Atlantic ‘S4 up-down’ and ‘S5 down-up’, however duration was shorter in Australia^48^.

The convex variant of Class-8 was notably similar to Norwegian ‘NPW 128’ and ‘NPW 129’^19^. Both vocalisations had an overall convex shape of approximately 0.06 s to 1 s duration, more than five harmonics (fundamental plus overtones) and one extremum (Fig. 2h)^19^. Class-9 was somewhat similar to Norwegian ‘simple burst pulse structure’ (Fig. (61)a in Ref.^19^); both vocalisations appeared as overall upsweeps (i.e., pulses occurred at increasing pulse-repetition rate), with seven harmonics. However, the two vocalisations varied slightly in duration and frequency range^19^. Class-11 (Fig. 2k) showed similarity to the second variation of ‘signal 1’ from the western Mediterranean population^50^. These vocalisations had an overall sine contour, harmonic overtones exceeding 14 kHz and a similar duration of approximately 0.5 s to 1.20 s^50^. Interestingly, Class-16 (Fig. 2p), a complex vocalisation that comprised three successive parts, was almost identical to ‘two inflected calls with click series component’ from northeast Atlantic long-finned pilot whales^37^. Both vocalisations showed similar components, contours, durations, and frequencies, although the northeast Atlantic vocalisation ended in a downsweep rather than a plateau.

Similarities between vocalisation contours across geographic locations could reflect lines of descent. Vocalisation characteristics, such as contour, have been shown to follow taxonomic lines, therefore animals that are genetically related will show more similar vocalisations^22,51^, even when populations are geographically separated. It has been postulated that the last overlap in home ranges of pilot whales was over 10,000 years, when the cooling of the ocean restricted home ranges to the tropics and subsequent warming allowed separation of the northern and southern hemisphere populations. Given such little time of geographic separation, the two populations may not yet have acquired distinctive vocalisations^52^. Similarities can also occur due to population mixing, enabling vocal influence, as reported in bottlenose dolphins^43,44^.

While greater vocal differences are expected in inter-species comparisons, southern Australian long-finned pilot whale vocalisations were comparable to Canary Island short-finned pilot whales (*Globicephala macrorhynchus*)^53^. One component from Canary Island ‘T-15iv’ respectively matched the frequency and duration measures of the convex variant of Class-8 (Fig. 2h)^53^. However, northern hemisphere reports indicate difficulty in distinguishing short-finned and long-finned pilot whales at sea^29^.

Further, Class-9 (Fig. 2i) was structurally similar to Australian killer whale ‘BC01’^54^. Both vocalisations comprised an upsweep with high frequency modulation, and similar frequency ranges^54^. Inter-species interactions are regularly observed in the wild^55^, some living in sympatry, such as pilot and killer whales^56,57^. Pilot whales exhibit vocal mimicry behaviour and have been shown to be attracted to killer whale vocalisations^55,58^. Consequently, southern Australian long-finned pilot whales may be mimicking Australian killer whale vocalisations in order to mask themselves, acting as an anti-predator mechanism, whilst allowing them to scavenge food remnants from killer whales. Whilst killer whales predating on long-finned pilot whales has not been documented in Australian waters, predation on other blackfish (i.e., false killer whales, *Pseudorca crassidens*) has been reported from New Zealand^59^. Predation risk might also lead to mobbing behaviour, as observed from pilot whales towards killer whales in Spain^57^.

Many biphonations and multi-component vocalisations were recorded from southern Australian long-finned pilot whales. The combination of multiple components may increase the likelihood of vocalisation recognition, crucial for long-distance communication in noisy environments^35,60^. Furthermore, in particular the graded and multi-component (including biphonations) classes 13–16 occurred in repeated sequences. These results are comparable to Canadian long-finned pilot whales^36^. The reasons behind such repetitiveness is currently unknown but could be an indicator that repetition also helps communication in noisy environments or maintains group cohesion^35,36^.

Southern Australian long-finned pilot whales further showed evidence of duetting. Duets are alternating or overlapping vocalisations produced by two individuals, whereby one individual is the initiator and the second individual is the responder^61^. The elements of a duet are typically repeated many times, such as the vocalisations in this study (Fig. 3e). Duetting is largely recorded in songbirds, insects, and primates with many hypotheses for its functionality, including sexual advertisement, mate-guarding, or defence of territories and resources^62–64^. Reports of duetting in cetaceans are rare. ‘Call-type matching’ has been documented in a group of killer whales, whereby different individuals contribute to sequences of stereotypical vocalisations^10^. As both sexes produce vocalisations and such vocalisations are not limited to reproductive activities, it is unlikely that duetting is a function of sexual advertisement in killer whales^10^. Rather, it was expected that duetting had a function of mediating group behaviour when members were dispersed. The role of duetting in pilot whales is unknown.

Our study showed that long-finned pilot whales off southern Australia share many call features with northern hemisphere pilot whales. Some of the stereotypical vocalisation classes recorded north of the equator were also found off Australia. Additional information on distribution, body size, genetics, and behaviour at times of recording may shed light upon the reasons behind vocalisation similarities and divergence within the species. Defining the vocal repertoire of a species is necessary for species acoustic identification, which enables PAM to be used as a tool for conservation management. Future sampling will be essential to determine population size, distribution, abundance, and trends, and to identify any impacts of anthropogenic stressors, such as underwater noise.

## Methods

### Data collection

**Figure 4:**
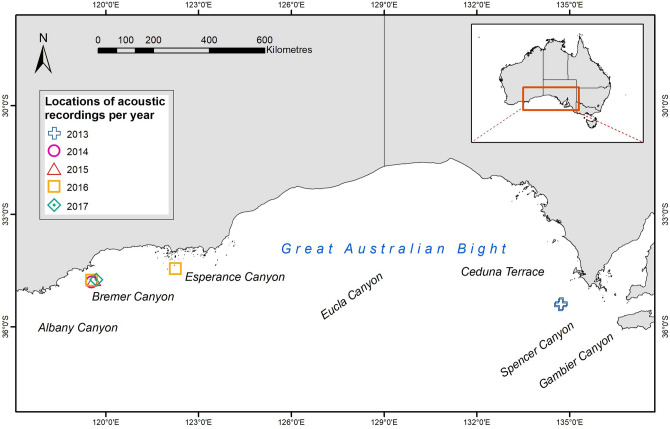
Map of the study region, southern coast of Australia, showing the locations where acoustic recordings were taken between 2013 and 2017.

Acoustic recordings of long-finned pilot whales were collected along the south coast of Australia between 2013 and 2017 (Fig. 4, Table 3). During every recording, information on pilot whale group size, composition, and behavioural state was noted. Surface behaviour was assigned to one of four behavioural states adapted from previous delphinid studies^9,36,65^: (1) travelling, (2) feeding, (3) milling, and (4) socialising. Environmental data were also collected during sightings, including sea-state, wind speed, swell height, and visibility. Only recordings obtained from single species sightings (i.e., long-finned pilot whales) were included in analyses.

**Table 3:** Metadata table for sample collections of long-finned pilot whale vocalisations.

Site	BSB	BSB	BSB	BSB	GAB	GAB	GAB
Latitude	− 34.8147	− 34.7563	− 34.7653	− 34.7536	− 35.3969	− 35.4327	− 34.4578
Longitude	119.5381	119.6247	119.5484	119.6930	134.7252	134.6512	122.2555
Date	26/02/14	17/02/15	9/02/16	31/03/17	01/05/13	06/05/13	10/01/16
Local recording start time	12:18	11:22	12:50	14:03	15:15	16:00	11:00
Cumulative recording duration	0:13:39	0:11:27	0:07:22	0:18:24	2:54:08	3:08:08	0:27:50
Number of files	1	1	1	1	3	3	270
Sampling frequency [kHz]	96	96	96	192	48	48	48
Amplitude resolution [bits]	24	24	24	24	16	16	16
Number of animals	60	20	60	50	10	60	20
Animal behaviour	Travelling	Milling	Socialising	Travelling	Travelling	Travelling	Travelling
Vocalisation classes present	Class-1 Class-2 Class-3 Class-4 Class-5 Class-6 Class-8 Class-9 Class-10 Class-11	Class-1 Class-2 Class-3 Class-4 Class-5 Class-6	Class-1 Class-2 Class-3 Class-4 Class-5 Class-6 Class-8 Class-10 Class-11 Class-12 Class-16	Class-1 Class-2 Class-3 Class-4 Class-5 Class-6 Class-8 Class-9 Class-11 Class-12 Class-15	Class-1 Class-2 Class-3 Class-4 Class-5 Class-6	Class-1 Class-2 Class-3 Class-4 Class-5 Class-6 Class-7 Class-8 Class-9 Class-12 Class-14	Class-1 Class-2 Class-3 Class-4 Class-5 Class-6 Class-8 Class-9 Class-12 Class-13 Class-17 Class-18
Data From	CMST	CMST	CMST	CMST	IFAW	IFAW	CWR

Locations: *BSB* Bremer Sub-Basin, *GAB* Great Australian Bight. Data owners: *CMST* Centre for Marine Science and Technology, *IFAW* International Fund for Animal Welfare, *CWR* Centre for Whale Research.

#### Eastern Great Australian Bight (GAB), South Australia, 2013

Acoustic recordings of long-finned pilot whales were collected during vessel-based line-transects in the eastern GAB between April and May 2013, covering a 15,130 km^2^ offshore area located to the south of Spencer Gulf and east of Kangaroo Island. Acoustic surveys were conducted under sail, motor, or motor/sail at 5–8 knots, a speed that allowed the hydrophone array to stream while reducing strum and excessive strain. Species identity was confirmed by two rotating observers with an eye height of approximately 5.6 m, scanning continuously during daylight hours in sea states below 4.

A 300-m hydrophone array was towed from the *SV Pelican* consisting of two low-frequency Benthos AQ4 elements (flat response within 1.5 dB from 10 Hz to 30 kHz) spaced 100 m apart and two high-frequency elements (flat response within 3 dB from 2 to 200 kHz) spaced 0.25 m apart. The pairs of hydrophones were used to obtain range and bearing information to vocalising animals. The two low-frequency hydrophones were primarily used to collect data on baleen whales, sperm whales (*Physeter macrocephalus*), and dolphins—including pilot whales, while the two high-frequency elements were used to detect beaked whales and pygmy or dwarf sperm whales (*Kogia* sp.). Continuous 16-bit stereo recordings were made with PAMGuard v1.12.05 (www.pamguard.org) at a sampling rate of 48,000 samples/s from the low-frequency elements, via a bespoke Seiche buffer box (Seiche Ltd., Devon, United Kingdom) passing signals to an RME Fireface sound card (RME Audio, Haimhausen, Germany). Recordings from the high-frequency elements were not used in the pilot whale study.

#### Bremer Sub-Basin (BSB), Western Australia, 2014–2017

Acoustic recordings of long-finned pilot whales were opportunistically collected in the BSB, during surveys focussing on killer whales in 2014, 2015, 2016, and 2017^54,66^. The BSB is located off the southwest continental shelf of Australia and extends over an area of 11,500 km^2^ in water depths of 100–4500 m^67^ (Fig. 4). The BSB forms part of the Albany Canyons group. Killer whales may occupy the area at any time of the year but have been found in abundance during the months of January to April^54,66^. While killer whales were the original target species for data collection, a large variety of marine megafauna were observed utilising this area, including long-finned pilot whales, sperm whales, and beaked whales^68^.

Acoustic recordings in the BSB were obtained with two devices. Primarily, recordings were collected by a HTI-96-MIN hydrophone (High Tech Inc., Long Beach, MS, USA) connected to a Sound Devices 722 digital recorder (Sound Devices Corp., Reedsburg, WI, USA), sampling at 96 kHz, 24 bit. The hydrophone was deployed overside during an encounter when the vessel was stationary. Secondarily, a self-contained underwater sound recorder (SoundTrap; Ocean Instruments, Auckland, New Zealand) was deployed overside during an encounter when the vessel was travelling less than 5 knots. The SoundTrap sampled at 192 kHz, 16 bit.

#### Western GAB, 2016

Acoustic recordings of long-finned pilot whales were opportunistically collected east of Esperance Canyon in the western GAB during a non-systematic survey in 2016. A 450-m hydrophone array was towed from the RV *Whale Song* at 6 knots during a 600-nm passage. The array consisted of two high-frequency elements (flat response within 1.5 dB from 20 to 44 kHz) spaced 0.25 m apart, and two mid-frequency elements (flat response within 1.5 dB from 10 to 44 kHz) spaced 2.84 m apart. Sound was sampled at 48 kHz (16 bits) from both mid- and high-frequency elements via a bespoke buffer box (Defence Science and Technology, Australia) and RME Fireface sound card. Real-time signal detection and recordings were made using PAMGuard v1.12.05, with a detection threshold set at 7 dB. The recordings were thus broken into many short files (i.e., 25).

### Data analysis

All recordings were examined in Raven Pro (Version 1.5; Bioacoustics Research Program, The Cornell Lab of Ornithology, Ithaca, NY, USA). Spectrograms were computed in Hann windows with 90% overlap (NFFT = 4096, 2048, or 1024 for fs = 192, 96, or 48 kHz, respectively). Vocalisations right at the start or end of a recording were ignored, as they could have been cut short by the recorder. Vocalisations were considered to have good signal-to-noise ratio if the entire contour was visible and no parts were masked by noise or fading in and out.

Some vocalisations consisted of multiple components. Biphonations were identified as two simultaneous but differently modulated components. They had to occur in the same arrangement (i.e., same relative sound levels of the two components and same relative timing) more than once to rule out that the components originated from two different animals. Similarly, vocalisations with multiple successive components had to occur more than once in the same arrangement (i.e., same succession, no gap in time, and same relative sound levels of the components) to be considered one vocalisation.

**Figure 5:**
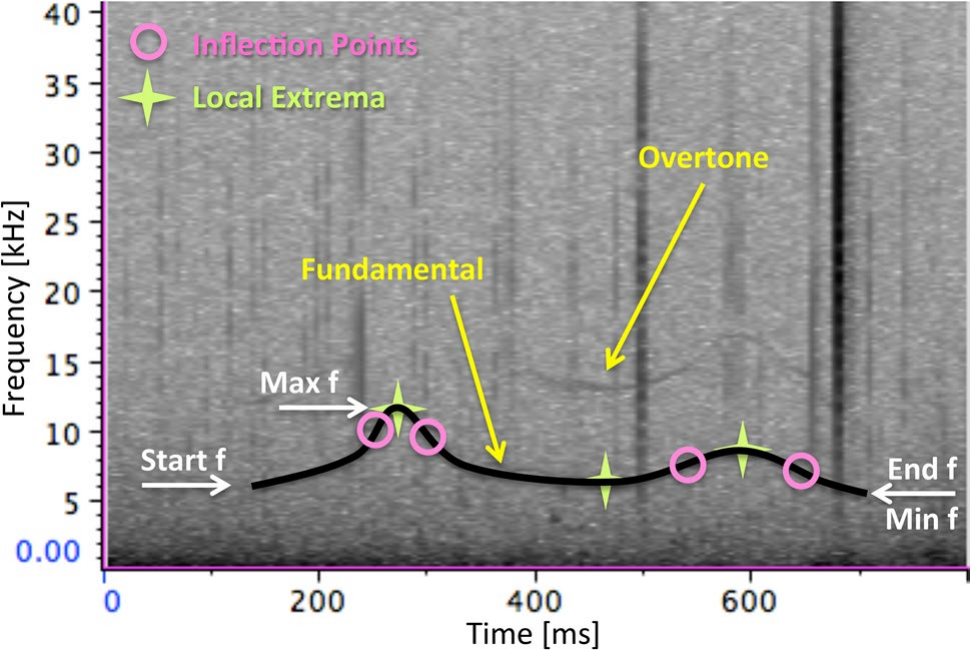
Example spectrogram indicating most of the parameters measured for vocalisations.

All components of multi-component vocalisations were measured individually. The following measurements were taken from the fundamental of all vocalisations: duration (Dur) [s], minimum frequency (Min f) [Hz], maximum frequency (Max f) [Hz], start frequency (Start f) [Hz], end frequency (End f) [Hz], number of local extrema (Extr) [n], number of inflection points (Infl) [n], number of steps (Step) [n], and number of overtones [n]. Figure 5 shows some of these measurements from an example vocalisation.

The minimum frequency is the lowest frequency of a contour, the maximum frequency is the highest frequency reached by the same contour. The start and end frequencies measure the frequencies at the beginning and end of a contour, respectively. Slope was computed as the ratio of ‘end frequency minus start frequency’ and duration. While some vocalisations were a tone of constant frequency, most vocalisations’ contours changed in frequency over the duration of the vocalisation. These vocalisations were frequency modulated. For example, upsweeps were modulated from lower to higher frequency over the duration of the vocalisation. If vocalisations were also amplitude-modulated (or, in the extreme case, pulsed), then sidebands appeared around the fundamental and around overtones (see examples in Class-6 and Class-18). These sidebands were not harmonically related to the fundamental contour. In the case of so-called burst-pulse sounds, contours seen in spectrograms are related to the pulse repetition rate; the faster the pulses, the higher the frequency. The pulse repetition rate can always be read off the spectrogram as the ‘harmonic interval’ between neighbouring contours^39^. Frequency modulation of the contours in burst-pulse sounds is related to changing pulse repetition rates.

A local extremum is a point at which the local slope of a contour (i.e., the first derivative of the contour with respect to time) changes from positive (i.e., rising) to negative (i.e., falling), or vice versa. The tangent to the contour is horizontal at all extrema. Local extrema can be either minima or maxima of the contour. At an inflection point, the curvature (i.e., the second derivative with respect to time) of the contour changes from clockwise to counterclockwise, or vice versa, and the tangent to the contour crosses the contour. Both extrema and inflection points can easily be determined using differential calculus^69^. Steps are discontinuities in frequency where the contour ‘jumps’ without any gap in time. For all measurements, means, standard deviations, medians, quartiles, and 10^th^ and 90^th^ percentiles were computed.

All vocalisations were further classified into contour classes, based on similarity of the lowest contour, frequency range (including overtones), and duration. The differentiation between constant-wave (‘flat’) contours and gently frequency-modulated contours (i.e., mild upsweeps and downsweeps) was determined based on slope. A slope of − 1000 to + 1000 Hz/s was accepted for the flat contour class. While the first author classified all vocalisations, the five co-authors independently classified a subset of 70 randomly selected vocalisations which were representatives of all 18 vocalisation classes. All observers were advised of the quantitative slope criterion. Inter-observer reliability was computed as the Fleiss’ kappa statistic^70^ and interpreted according to Landis and Koch^71^.

The repetition of vocalisations of the same class was assessed one recorded file at a time. For every vocalisation in that file, the class of the next vocalisation was noted. Histograms were created for each class, showing how often it was immediately followed by each class. The percentage of times that each class was followed by a vocalisation of the same class was computed, as was the median time between two vocalisations of the same class.

Finally, the vocalisations of southern Australian long-finned pilot whales were compared to those of pilot whales elsewhere by comparing spectrograms and measurements with those found in the literature.

## Supplementary information

Supplementary Information.

## Data Availability

The long-finned pilot whale vocalisations of this article are available in WAV format for download from Dryad (10.5061/dryad.w3r2280p3).
