# Supplementary material for: Australian long-finned pilot whales (Globicephala melas) emit stereotypical, variable, biphonic, multi-component, and sequenced vocalisations, similar to those recorded in the northern hemisphere
Source: Sci Rep. 2020 Nov 26;10:20609. doi: 10.1038/s41598-020-74111-y (PMC7693278; doi:10.1038/s41598-020-74111-y)
Supplement: Supplementary file 1 — Supplementary Information. [file 41598_2020_74111_MOESM1_ESM.pdf]

Australian long-finned pilot whales (*Globicephala melas*) emit stereotypical, variable, biphonic, multi-component, and sequenced vocalisations, similar to those recorded in the northern hemisphere

Rachael Courts[1*], Christine Erbe[1], Rebecca Wellard[1,2], Oliver Boisseau[3], K. Curt Jenner[4], Micheline-N. Jenner[4]

[1] Centre for Marine Science and Technology, Curtin University, Perth, WA 6102, Australia

[2] Project ORCA, Perth, WA 6026, Australia

[3] *Song of the Whale* research team, Marine Conservation Research, 94 High Street, Kelvedon Essex CO5 9AA, United Kingdom

[4] Centre for Whale Research (WA) Inc., PO Box 1622, Fremantle, WA 6959, Australia

[*]19872816@student.curtin.edu.au

SUPPLEMENTARY INFORMATION

Tables S1-S18 summarise the measurements of all vocalisation classes.

Supplementary Table S1. Measurement summary of Class-1 (n = 654) fundamental contours.

	Dur (s)	Min f (Hz)	Max f (Hz)	Start f (Hz)	End f (Hz)	Extr (n)	Infl (n)	Step (n)	Overtone (n)
Mean	0.40	4707	6716	4765	6634	0.0	0.3	0.3	0.5
Standard Deviation	0.25	2468	3143	2457	3166	0.2	0.6	1.1	0.9
Minimum	0.03	702	1480	711	1225	0.0	0.0	0.0	0.0
Maximum	1.67	14041	19092	14045	19089	3.0	9.0	9.0	4.0
10th Percentile	0.11	2211	3123	2234	2974	0.0	0.0	0.0	0.0
25th Percentile	0.19	2785	4113	2829	3994	0.0	0.0	0.0	0.0
50th Percentile	0.37	4185	6154	4224	6089	0.0	0.0	0.0	0.0
75th Percentile	0.56	6072	8844	6187	8780	0.0	0.0	0.0	1.0
90th Percentile	0.73	8008	10888	8043	10863	0.0	1.0	1.0	2.0

Supplementary Table S2. Measurement summary of Class-2 (n = 488) fundamental contours.

	Dur (s)	Min f (Hz)	Max f (Hz)	Start f (Hz)	End f (Hz)	Extr (n)	Infl (n)	Step (n)	Overtone (n)
Mean	0.43	4090	5454	5323	4151	0.1	0.2	0.1	0.5
Standard Deviation	0.28	2286	2747	2726	2310	0.5	0.6	0.6	0.9
Minimum	0.03	445	954	656	525	0.0	0.0	0.0	0.0
Maximum	2.07	14259	18276	18270	14259	5.0	5.0	10.0	4.0
10th Percentile	0.12	1800	2715	2523	1850	0.0	0.0	0.0	0.0
25th Percentile	0.20	2500	3393	3301	2542	0.0	0.0	0.0	0.0
50th Percentile	0.36	3340	4639	4549	3409	0.0	0.0	0.0	0.0
75th Percentile	0.59	5082	7071	6828	5150	0.0	0.0	0.0	1.0
90th Percentile	0.82	7288	9277	8992	7335	0.0	1.0	0.0	2.0

Supplementary Table S3. Measurement summary of Class-3 (n = 316) fundamental contours.

	Dur (s)	Min f (Hz)	Max f (Hz)	Start f (Hz)	End f (Hz)	Extr (n)	Infl (n)	Step (n)	Overtone (n)
Mean	0.44	3806	4331	4038	4024	0.1	0.0	0.1	0.5
Standard Deviation	0.31	2329	2456	2407	2389	0.3	0.2	0.4	0.9
Minimum	0.05	569	919	700	700	0.0	0.0	0.0	0.0
Maximum	1.98	14289	16615	16613	14351	2.0	1.0	3.0	4.0
10th Percentile	0.10	1828	2177	1970	1968	0.0	0.0	0.0	0.0
25th Percentile	0.18	2381	2799	2530	2537	0.0	0.0	0.0	0.0
50th Percentile	0.38	3017	3525	3269	3162	0.0	0.0	0.0	0.0
75th Percentile	0.64	4448	4908	4575	4713	0.0	0.0	0.0	1.0
90th Percentile	0.86	7300	8044	7781	7698	0.0	0.0	0.0	2.0

Supplementary Table S4. Measurement summary of Class-4 (n = 58) fundamental contours

	Dur (s)	Min f (Hz)	Max f (Hz)	Start f (Hz)	End f (Hz)	Extr (n)	Infl (n)	Step (n)	Overtone (n)
Mean	0.61	5346	7524	6504	6217	2.7	2.6	0.1	0.4
Standard Deviation	0.37	3004	3636	3354	3386	1.2	1.4	0.5	0.6
Minimum	0.06	739	2629	1706	872	1.0	0.0	0.0	0.0
Maximum	1.51	13113	18443	14644	16113	7.0	8.0	3.0	2.0
10th Percentile	0.16	2003	3730	2879	2671	2.0	1.3	0.0	0.0
25th Percentile	0.31	3247	4123	3788	3508	2.0	2.0	0.0	0.0
50th Percentile	0.57	4520	7332	5396	5666	2.0	2.0	0.0	0.0
75th Percentile	0.79	7426	9754	8706	8400	3.0	3.0	0.0	1.0
90th Percentile	1.23	10089	12572	11151	10481	4.7	4.7	0.0	1.0

Supplementary Table S5. Measurement summary of Class-5 (n = 159) fundamental contours.

	Dur (s)	Min f (Hz)	Max f (Hz)	Start f (Hz)	End f (Hz)	Extr (n)	Infl (n)	Step (n)	Overtone (n)
Mean	0.32	4569	5850	5512	5579	1.0	0.1	0.2	0.7
Standard Deviation	0.27	3199	3609	3423	3582	0.2	0.3	1.1	1.0
Minimum	0.06	1006	1546	1400	1400	1.0	0.0	0.0	0.0
Maximum	1.20	14685	18708	16516	18700	3.0	3.0	9.0	4.0
10th Percentile	0.10	1361	2041	1938	1856	1.0	0.0	0.0	0.0
25th Percentile	0.14	1939	3164	2891	3078	1.0	0.0	0.0	0.0
50th Percentile	0.20	3762	5048	4743	4608	1.0	0.0	0.0	0.0
75th Percentile	0.43	6525	7870	7805	7471	1.0	0.0	0.0	1.0
90th Percentile	0.71	9732	11399	10576	11396	1.0	0.0	0.0	2.0

Supplementary Table S6. Measurement summary of Class-6 (n = 111) fundamental contours.

	Dur (s)	Min f (Hz)	Max f (Hz)	Start f (Hz)	End f (Hz)	Extr (n)	Infl (n)	Step (n)	Overtone (n)
Mean	0.38	4552	5711	4821	4782	1.0	0.1	0.1	0.3
Standard Deviation	0.29	2715	3004	2881	2703	0.1	0.4	0.7	0.6
Minimum	0.05	1423	2320	1454	1665	1.0	0.0	0.0	0.0
Maximum	1.30	12619	17058	16100	12967	2.0	2.0	7.0	3.0
10th Percentile	0.10	1820	2640	1939	1960	1.0	0.0	0.0	0.0
25th Percentile	0.16	2526	3517	2779	2835	1.0	0.0	0.0	0.0
50th Percentile	0.27	3323	4416	3662	3630	1.0	0.0	0.0	0.0
75th Percentile	0.54	6101	7738	6703	6608	1.0	0.0	0.0	0.0
90th Percentile	0.82	8551	9927	8923	8709	1.0	0.0	0.0	1.0

Supplementary Table S7. Measurement summary of Class-7 (n = 28) fundamental contours.

	Dur (s)	Min f (Hz)	Max f (Hz)	Start f (Hz)	End f (Hz)	Extr (n)	Infl (n)	Step (n)	Overtone (n)
Mean	0.63	3202	5015	3403	4867	0.1	0.5	0.0	0.0
Standard Deviation	0.12	238	503	361	615	0.3	0.6	0.0	0.0
Minimum	0.42	2712	4307	2931	3762	0.0	0.0	0.0	0.0
Maximum	0.84	3652	6211	4286	6208	1.0	2.0	0.0	0.0
10th Percentile	0.48	2931	4354	2974	3927	0.0	0.0	0.0	0.0
25th Percentile	0.56	3017	4593	3108	4454	0.0	0.0	0.0	0.0
50th Percentile	0.62	3149	5014	3355	4885	0.0	0.0	0.0	0.0
75th Percentile	0.71	3434	5336	3637	5183	0.0	1.0	0.0	0.0
90th Percentile	0.82	3498	5717	3939	5714	0.7	1.0	0.0	0.0

Supplementary Table S8. Measurement summary of all Class-8 (n = 73) variants

	Dur (s)	Min f (Hz)	Max f (Hz)	Start f (Hz)	End f (Hz)	Extr (n)	Infl (n)	Step (n)	Overtone (n)
Mean	0.31	1830	2914	2659	2175	0.2	0.1	0.0	9.4
Standard Deviation	0.21	1180	1361	1408	1225	0.4	0.4	0.0	6.4
Minimum	0.06	349	1014	742	350	0.0	0.0	0.0	5.0
Maximum	1.15	5900	7918	7915	5903	1.0	2.0	0.0	41.0
10th Percentile	0.11	747	1384	1293	776	0.0	0.0	0.0	5.0
25th Percentile	0.16	1015	1940	1621	1137	0.0	0.0	0.0	5.0
50th Percentile	0.27	1393	2594	2263	2010	0.0	0.0	0.0	7.0
75th Percentile	0.40	2527	3594	3232	2842	0.3	0.0	0.0	11.0
90th Percentile	0.55	3244	4836	4615	3835	1.0	0.0	0.0	16.2

Supplementary Table S9. Measurement summary of Class-9 (n = 19) fundamental contours.

	Dur (s)	Min f (Hz)	Max f (Hz)	Start f (Hz)	End f (Hz)	Extr (n)	Infl (n)	Step (n)	Overtone (n)
Mean	0.43	2243	4741	2326	4636	0.0	0.1	0.9	7.9
Standard Deviation	0.26	1106	2445	1069	2429	0.0	0.2	1.7	6.9
Minimum	0.06	624	2051	858	1858	0.0	0.0	0.0	3.0
Maximum	1.06	4524	11145	4525	10924	0.0	1.0	5.0	36.0
10th Percentile	0.14	922	2610	1034	2550	0.0	0.0	0.0	5.0
25th Percentile	0.22	1350	2876	1367	2783	0.0	0.0	0.0	5.0
50th Percentile	0.34	2103	3526	2106	3300	0.0	0.0	0.0	6.0
75th Percentile	0.61	2738	5761	2769	5703	0.0	0.0	1.0	7.8
90th Percentile	0.82	4209	8341	4276	8186	0.0	0.0	4.2	10.2

Supplementary Table S10. Measurement summary of Class-10 (n = 11) fundamental contours

	Dur (s)	Min f (Hz)	Max f (Hz)	Start f (Hz)	End f (Hz)	Extr (n)	Infl (n)	Step (n)	Overtone (n)
Mean	0.69	1645	7842	1844	6014	0.1	0.5	0.5	6.2
Standard Deviation	0.16	953	2728	1045	2755	0.3	0.7	1.0	1.3
Minimum	0.36	828	3340	855	2403	0.0	0.0	0.0	5.0
Maximum	0.88	3531	11034	3862	10924	1.0	2.0	3.0	9.0
10th Percentile	0.46	851	3409	919	2677	0.0	0.0	0.0	5.0
25th Percentile	0.57	954	5610	1126	3322	0.0	0.0	0.0	5.0
50th Percentile	0.67	1225	8351	1338	6431	0.0	0.0	0.0	6.0
75th Percentile	0.84	2261	10300	2495	7268	0.0	1.0	0.0	7.0
90th Percentile	0.88	3399	10814	3796	10527	0.4	1.4	2.4	8.4

Supplementary Table S11. Measurement summary of Class-11 (n = 3) fundamental contours.

	Dur (s)	Min f (Hz)	Max f (Hz)	Start f (Hz)	End f (Hz)	Extr (n)	Infl (n)	Step (n)	Overtone (n)
Mean	0.96	4218	9036	4512	7805	3.7	4.7	0.0	6.0
Standard Deviation	0.22	1893	4806	1498	4473	0.5	0.5	0.0	1.4
Minimum	0.74	1545	2786	2400	1600	3.0	4.0	0.0	5.0
Maximum	1.26	5691	14474	5706	11972	4.0	5.0	0.0	8.0
50th Percentile	0.89	5418	9846	5430	9843	4.0	5.0	0.0	5.0

Supplementary Table S12. Measurement summary of Class-12 (n = 13) fundamental contours.

	Dur (s)	Min f (Hz)	Max f (Hz)	Start f (Hz)	End f (Hz)	Extr (n)	Infl (n)	Step (n)	Overtone (n)
Mean	0.82	1783	2621	2004	2234	0.1	0.1	0.1	11.5
Standard Deviation	0.27	860	1066	909	1043	0.3	0.3	0.3	11.7
Minimum	0.52	433	693	433	550	0.0	0.0	0.0	5.0
Maximum	1.70	3495	4549	3588	4243	1.0	1.0	1.0	50.0
10th Percentile	0.61	943	1475	1136	1208	0.0	0.0	0.0	5.0
25th Percentile	0.70	1234	1907	1399	1423	0.0	0.0	0.0	5.8
50th Percentile	0.75	1299	2258	1627	1979	0.0	0.0	0.0	6.0
75th Percentile	0.86	2347	3549	3000	3147	0.0	0.0	0.0	13.5
90th Percentile	1.10	3074	4043	3363	3719	0.2	0.2	0.2	22.8

Supplementary Table S13. Measurement summary of Class-13 (n = 3) fundamental contours.

	Dur (s)	Min f (Hz)	Max f (Hz)	Start f (Hz)	End f (Hz)	Extr (n)	Infl (n)	Step (n)	Overtone (n)
Mean	1.16	567	5134	770	4658	1.3	1.3	0.0	3.0
Standard Deviation	0.13	102	1529	305	1643	0.5	0.5	0.0	0.8
Minimum	1.01	495	3650	495	2722	1.0	1.0	0.0	2.0
Maximum	1.33	712	7237	1196	6739	2.0	2.0	0.0	4.0
50th Percentile	1.13	495	4516	619	4514	1.0	1.0	0.0	3.0

Supplementary Table S14. Measurement summary of Part 1 and Part 2 of Class-14 biphonations (n = 48).

	Dur (s)	Min f (Hz)	Max f (Hz)	Start f (Hz)	End f (Hz)	Extr (n)	Infl (n)	Step (n)	Overtone (n)
Part 1									
Mean	0.61	2364	3888	3756	2769	0.0	0.0	0.2	2.1
Standard Deviation	0.18	886	1548	1553	1154	0.0	0.0	0.5	1.7
Minimum	0.24	831	1181	1050	1050	0.0	0.0	0.0	0.0
Maximum	0.96	5139	6823	6648	6473	0.0	0.0	3.0	5.0
10th Percentile	0.37	1575	2100	1937	1589	0.0	0.0	0.0	0.0
25th Percentile	0.46	1662	2231	2186	1881	0.0	0.0	0.0	1.0
50th Percentile	0.68	2362	3827	3673	2647	0.0	0.0	0.0	1.0
75th Percentile	0.73	2602	5358	5205	3149	0.0	0.0	0.0	4.0
90th Percentile	0.84	2731	5848	5837	4289	0.0	0.0	1.0	5.0
Part 2									
Mean	0.44	8502	11033	8521	10962	0.0	0.1	0.2	0.0
Standard Deviation	0.21	2707	2638	2698	2678	0.0	0.4	0.8	0.1
Minimum	0.17	4175	6588	4179	6585	0.0	0.0	0.0	0.0
Maximum	1.04	13122	15877	13122	15874	0.0	1.0	4.0	1.0
10th Percentile	0.23	5594	8688	5609	8488	0.0	0.0	0.0	0.0
25th Percentile	0.28	6911	9103	6912	9067	0.0	0.0	0.0	0.0
50th Percentile	0.40	7173	9710	7218	9685	0.0	0.0	0.0	0.0
75th Percentile	0.49	12041	14281	12044	14278	0.0	0.0	0.0	0.0
90th Percentile	0.80	12575	14866	12575	14864	0.0	1.0	0.0	0.0

Supplementary Table S15. Measurement summary of Part 1 and Part 2 of Class-15 biphonations (n = 16).

	Dur (s)	Min f (Hz)	Max f (Hz)	Start f (Hz)	End f (Hz)	Extr (n)	Infl (n)	Step (n)	Overtone (n)
Part 1									
Mean	0.89	715	4008	1778	821	0.0	0.1	0.0	6.4
Standard Deviation	0.11	421	890	1050	408	0.0	0.2	0.0	1.3
Minimum	0.63	219	1741	580	310	0.0	0.0	0.0	5.0
Maximum	1.08	1969	4862	3916	1973	0.0	1.0	0.0	8.0
10th Percentile	0.77	310	2150	622	317	0.0	0.0	0.0	5.0
25th Percentile	0.81	413	3852	1002	501	0.0	0.0	0.0	5.0
50th Percentile	0.90	594	4199	1282	866	0.0	0.0	0.0	6.5
75th Percentile	0.96	869	4705	2838	1002	0.0	0.0	0.0	8.0
90th Percentile	1.02	1201	4787	3296	1210	0.0	0.0	0.0	8.0
Part 2									
Mean	0.43	8625	17230	8634	17223	0.0	0.3	0.6	2.5
Standard Deviation	0.13	1795	3754	1793	3753	0.0	0.4	0.5	2.4
Minimum	0.26	4739	12184	4740	12174	0.0	0.0	0.0	0.0
Maximum	0.62	11618	24787	11624	24781	0.0	1.0	1.0	7.0
10th Percentile	0.29	5188	12339	5198	12338	0.0	0.0	0.0	0.0
25th Percentile	0.30	7856	12945	7896	12942	0.0	0.0	0.0	0.0
50th Percentile	0.43	8913	18709	8914	18683	0.0	0.0	1.0	2.0
75th Percentile	0.57	9632	19877	9635	19872	0.0	0.5	1.0	5.0
90th Percentile	0.60	10617	20661	10620	20658	0.0	1.0	1.0	5.9

Supplementary Table S16. Measurement summary of Part 1, Part 2 and Part 3 of Class-16 biphonations (n = 13).

	Dur (s)	Min f (Hz)	Max f (Hz)	Start f (Hz)	End f (Hz)	Extr (n)	Infl (n)	Step (n)	Overtone (n)
Part 1									
Mean	0.29	1059	5627	1300	5620	0.2	0.0	0.0	5.5
Standard Deviation	0.08	396	1027	1002	1029	0.4	0.0	0.0	0.6
Minimum	0.19	431	4577	433	4575	0.0	0.0	0.0	5.0
Maximum	0.47	1740	7825	4505	7823	1.0	0.0	0.0	7.0
10th Percentile	0.19	506	4660	507	4656	0.0	0.0	0.0	5.0
25th Percentile	0.23	695	5041	696	5039	0.0	0.0	0.0	5.0
50th Percentile	0.27	1113	5258	1115	5255	0.0	0.0	0.0	5.0
75th Percentile	0.33	1421	5769	1504	5767	0.3	0.0	0.0	6.0
90th Percentile	0.41	1585	7758	2294	7756	1.0	0.0	0.0	6.2
Part 2									
Mean	0.24	11781	17873	11787	17852	0.0	0.0	2.2	2.0
Standard Deviation	0.05	1592	1638	1584	1677	0.0	0.0	0.8	3.7
Minimum	0.16	8969	14536	9031	14289	0.0	0.0	0.0	0.0
Maximum	0.37	14165	20969	14166	20965	0.0	0.0	3.0	10.0
10th Percentile	0.21	10329	16268	10341	16218	0.0	0.0	1.6	0.0
25th Percentile	0.22	10763	16856	10765	16853	0.0	0.0	2.0	0.0
50th Percentile	0.23	10887	17273	10887	17270	0.0	0.0	2.0	0.0
75th Percentile	0.25	13585	19018	13586	19012	0.0	0.0	3.0	1.8
90th Percentile	0.30	13769	20380	13771	20379	0.0	0.0	3.0	9.2
Part 3									
Mean	0.50	978	3675	1188	3648	0.0	0.0	0.0	7.4
Standard Deviation	0.17	408	957	645	965	0.0	0.0	0.0	2.3
Minimum	0.38	402	1794	405	1792	0.0	0.0	0.0	5.0
Maximum	0.96	1864	6080	2969	6078	0.0	0.0	0.0	13.0
10th Percentile	0.38	476	2913	577	2909	0.0	0.0	0.0	5.0
25th Percentile	0.39	711	3309	778	3216	0.0	0.0	0.0	5.8
50th Percentile	0.43	944	3402	965	3339	0.0	0.0	0.0	7.0
75th Percentile	0.53	1103	4096	1397	4085	0.0	0.0	0.0	9.0
90th Percentile	0.80	1723	4927	2086	4925	0.0	0.0	0.0	10.6

Supplementary Table S17. Measurement summary of Part 1, Part 2 and Part 3 of Class-17 biphonations (n = 8).

	Dur (s)	Min f (Hz)	Max f (Hz)	Start f (Hz)	End f (Hz)	Extr (n)	Infl (n)	Step (n)	Overtone (n)
Part 1									
Mean	0.14	4600	6010	5557	5936	1.0	0.0	0.0	2.9
Standard Deviation	0.01	263	674	499	711	0.0	0.0	0.0	1.3
Minimum	0.12	4021	4546	4546	4485	1.0	0.0	0.0	2.0
Maximum	0.17	4949	6619	6155	6616	1.0	0.0	0.0	6.0
25th Percentile	0.14	4510	5699	5299	5519	1.0	0.0	0.0	2.0
50th Percentile	0.14	4614	6239	5621	6161	1.0	0.0	0.0	2.5
75th Percentile	0.15	4790	6517	5959	6514	1.0	0.0	0.0	3.0
Part 2									
Mean	0.61	1245	1718	1424	1463	0.0	0.0	0.0	6.3
Standard Deviation	0.16	419	478	417	487	0.0	0.0	0.0	1.1
Minimum	0.38	1050	1392	1113	1113	0.0	0.0	0.0	5.0
Maximum	0.90	2351	2753	2412	2505	0.0	0.0	0.0	8.0
25th Percentile	0.48	1051	1411	1178	1178	0.0	0.0	0.0	5.5
50th Percentile	0.62	1088	1464	1247	1203	0.0	0.0	0.0	6.0
75th Percentile	0.71	1141	1925	1509	1662	0.0	0.0	0.0	7.0
Part 3									
Mean	0.25	8901	12160	8902	12158	0.0	0.4	0.0	0.3
Standard Deviation	0.06	768	203	768	203	0.0	0.5	0.0	0.4
Minimum	0.17	7567	11809	7568	11807	0.0	0.0	0.0	0.0
Maximum	0.36	9837	12378	9839	12377	0.0	1.0	0.0	1.0
25th Percentile	0.21	8348	11974	8349	11972	0.0	0.0	0.0	0.0
50th Percentile	0.24	9103	12245	9105	12242	0.0	0.0	0.0	0.0
75th Percentile	0.29	9450	12327	9452	12324	0.0	1.0	0.0	0.5

Supplementary Table S18. Measurement summary of Part 1, Part 2 and Part 3 of Class-18 biphonations (n = 7).

	Dur (s)	Min f (Hz)	Max f (Hz)	Start f (Hz)	End f (Hz)	Extr (n)	Infl (n)	Step (n)	Overtone (n)
Part 1									
Mean	0.14	3781	7089	3823	7080	0.0	0.0	0.0	2.7
Standard Deviation	0.03	501	915	481	910	0.0	0.0	0.0	1.3
Minimum	0.09	3247	5227	3249	5224	0.0	0.0	0.0	1.0
Maximum	0.20	4701	8017	4702	8011	0.0	0.0	0.0	5.0
25th Percentile	0.11	3358	6541	3412	6540	0.0	0.0	0.0	2.0
50th Percentile	0.13	3526	7485	3743	7482	0.0	0.0	0.0	2.0
75th Percentile	0.16	4174	7633	4176	7597	0.0	0.0	0.0	3.8
Part 2									
Mean	0.79	2232	2827	2394	2490	0.0	0.0	0.0	10.0
Standard Deviation	0.14	266	354	228	344	0.0	0.0	0.0	5.7
Minimum	0.62	1732	2412	2103	2041	0.0	0.0	0.0	0.0
Maximum	1.07	2646	3508	2831	3015	0.0	0.0	0.0	20.0
25th Percentile	0.67	2112	2565	2227	2238	0.0	0.0	0.0	6.8
50th Percentile	0.78	2227	2685	2370	2431	0.0	0.0	0.0	10.0
75th Percentile	0.84	2391	3070	2533	2845	0.0	0.0	0.0	12.8
Part 3									
Mean	0.62	8614	11907	11428	9044	0.7	0.7	0.0	0.9
Standard Deviation	0.18	1526	1591	1665	1990	0.7	0.7	0.0	0.6
Minimum	0.32	7670	10021	9495	7972	0.0	0.0	0.0	0.0
Maximum	0.92	12247	15526	15093	13856	2.0	2.0	0.0	2.0
25th Percentile	0.49	7821	11215	10276	8046	0.0	0.0	0.0	0.3
50th Percentile	0.64	7918	11529	11258	8103	1.0	1.0	0.0	1.0
75th Percentile	0.74	8626	11923	11530	8804	1.0	1.0	0.0	1.0

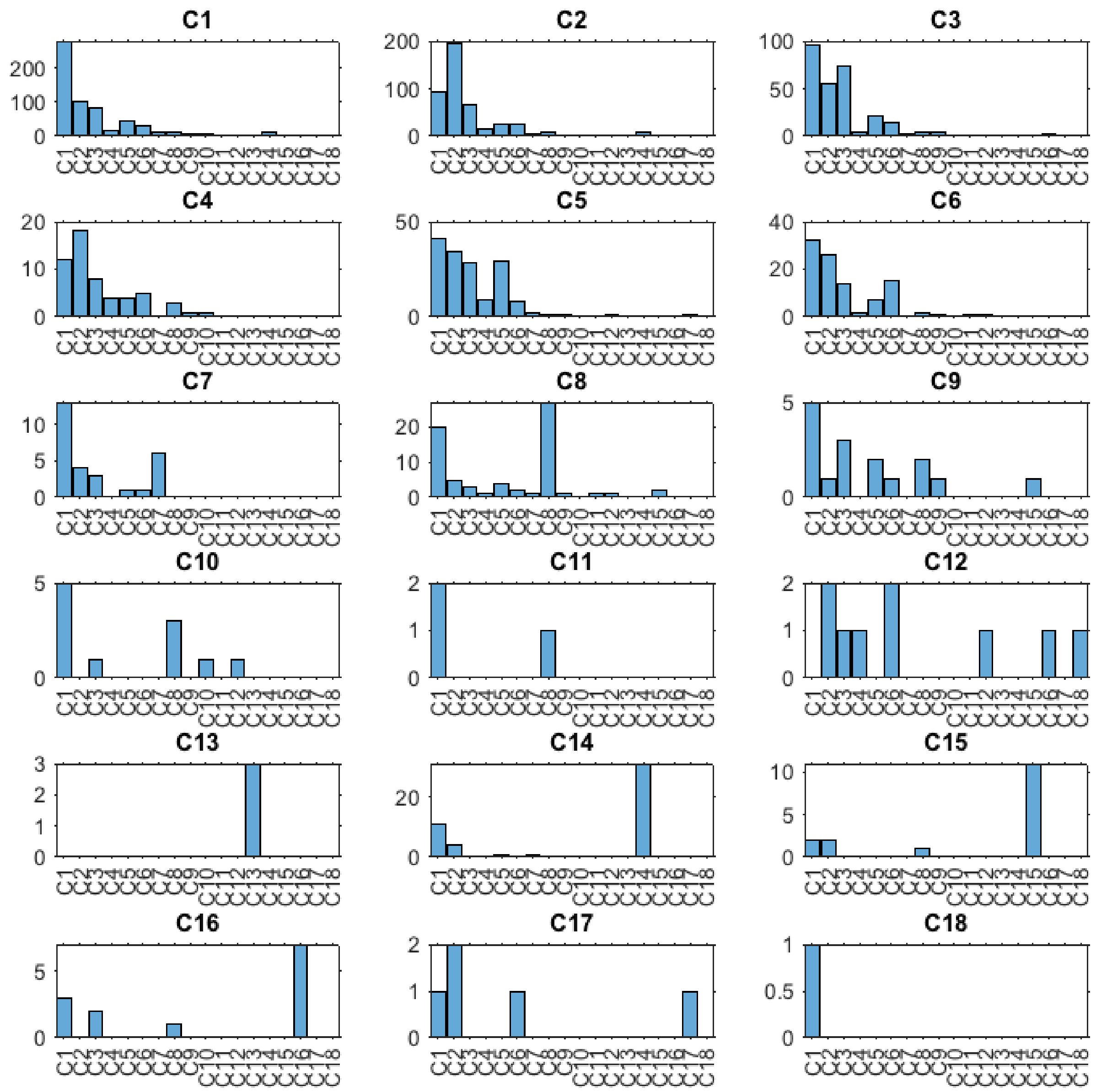

Supplementary Figure S1. Histograms of the numbers of times a vocalisation from each class was followed by a vocalisation from all other classes.
